# Mediterranean Diet and Cardiovascular Prevention: Why Analytical Observational Designs Do Support Causality and Not Only Associations

**DOI:** 10.3390/ijerph192013653

**Published:** 2022-10-21

**Authors:** Miguel Ángel Martínez-González, Nerea Martín-Calvo, Telmo Bretos-Azcona, Silvia Carlos, Miguel Delgado-Rodríguez

**Affiliations:** 1 Department of Preventive Medicine and Public Health, School of Medicine, University of Navarra, 31008 Pamplona, Spain; 2 IdiSNA, Navarra Institute for Health Research, 31008 Pamplona, Spain; 3 CIBER Fisiopatología de la Obesidad y Nutrición, 28029 Madrid, Spain; 4 Department of Health Sciences, University of Jaén, Área de Medicina Preventiva y Ciencias de la Salud, 23071 Jaén, Spain; 5 CIBER Epidemiología y Salud Pública, 28029 Madrid, Spain

**Keywords:** causal inference, Mediterranean diet, observational studies, risk assessment, statistical modeling of disease risk

## Abstract

Causal reductions in cardiovascular disease (CVD) with the Mediterranean diet (MedDiet) are supported by randomized trials, but the ability of nonrandomized studies to provide causal inferences in nutritional epidemiology is questioned. The “Seguimiento Universidad de Navarra” (SUN) project, conducted during 1999–2019 with 18,419 participants, was used to try to refute non-causal explanations for the inverse association found between adherence to the MedDiet and reduced CVD risk. A framework of different analytical strategies is proposed: alternative definitions of the exposure, exploration of residual confounding, resampling methods, depiction of absolute risks across the follow-up period, trial emulation, and negative controls. Additionally, we calculated the rate advancement period (RAP). We found that one standard deviation increase in the most frequently used MedDiet score was associated with a 29% relative reduction in CVD risk (95% Confidence Interval [CI] 14–41%), which is almost identical to that found in 2 randomized trials. The RAP of CVD would be postponed by an average of 7.9 years (95% CI: 1.6 to 14.2 years) by switching from low (MDS = 0 to2) to high (MDS = 7 to 9) adherence to the MedDiet in the fully adjusted model. Sensitivity analyses, graphical representations of absolute risks, trial emulation, and negative controls also supported causality. In conclusion, a framework of analytical approaches supported the causal effect of the MedDiet on CVD prevention using observational data. Similar methodology could be applied for causal inferences regarding other hypotheses.

## 1. Introduction

Cardiovascular disease (CVD) is the leading cause of nutrition-related deaths (10 million deaths) and disability adjusted life years (DALYs) (207 million) [1,2], and consequently, it is largely preventable. A huge body of knowledge has provided strong evidence in the last 2–3 decades to convincingly support ideal dietary approaches for CVD prevention. This evidence gives priority to the focus on overall dietary patterns, rather than on individual nutrients or supplements [3]. In this context, the traditional Mediterranean diet (MedDiet) is the model that probably has accrued the largest body of evidence supporting substantial benefits for CVD prevention [4,5,6,7,8,9,10,11,12,13,14,15,16,17,18,19,20,21,22]. 

Notwithstanding, the whole field of science pertaining to nutritional epidemiology has been questioned, particularly because of its exclusive reliance on mere observational designs [23,24]. Indeed, randomized controlled trials (RCTs) provide the strongest proof of causality and the aim of statistical methods applied to observational studies is not to replace RCTs. Nevertheless, RCTs are not always ethical or feasible and the main high-quality evidence available for many fields of public health should be based in well-designed and well-conducted cohort studies. In addition, some of the criticisms of these observational designs may be unfounded [25,26,27,28,29]. The ability of observational designs to support causal relationships has founded important actions in public health, as it has been the case for smoking and other relevant exposures. The aim of analytical observational studies is also to obtain causal inferences, not only ‘associations’. This goal is attained by means of good control of confounding, which is the hallmark of robust epidemiologic studies. Otherwise, investigators would present only non-adjusted estimates in observational studies. Reasonable control of confounding can be feasible with careful study design and thorough analyses [29]. In addition, the benefits of real-world prospective observational studies are unquestionable, including evaluation of real-world patients, detection of less common associations, and evaluation of under-studied and under-enrolled subpopulations.

The emerging literature including that relating to RCTs [4,28,30,31,32,33,34] shows sufficient evidence to support a causal inference of the beneficial cardiovascular effects of a MedDiet. Therefore, high-quality data already exist questioning the need for further studies other than RCTs. Given this unequivocal causal relationship between the MedDiet and CVD, there is an excellent opportunity to also test the ability of an observational design to provide causal answers to this same question and to obtain responses analogous to those attained from the main RCT, namely the PREDIMED trial [4]. Accepting that observational studies could be affected by limitations such as residual confounding and measurement errors and, therefore, cannot definitely prove causation [28,29], we used an observational design and applied different analytical approaches to attain a reasonable conclusion that such non-causal alternatives are very unlikely. 

In this context, we performed several analyses using data from the “Seguimiento Universidad de Navarra” (SUN) cohort [34,35] to answer the following seven questions: (1) Is the association dependent on the specific definition used to classify participants according to their adherence to the MedDiet? (2) Is the association dependent on exclusively one or only a few components of the MedDiet? (3) Is the association explained by better health consciousness or other beneficial lifestyles of those participants who are more adherent to the MedDiet (i.e., by residual confounding)? (4) Is the association related to particular characteristics of the assessed cohort (i.e., the specific analytical sample)? (5) Would the association be replicated in the emulation of a hypothetical target trial analogous to the largest available randomized trial (the PREDIMED trial)? (6) Beyond relative risks, what would be the absolute risks of adherence and non-adherence to the MedDiet throughout the follow-up time? (7) Is the association the result of any other artifact or bias that would also be apparent for other biologically less plausible outcomes?

## 2. Materials and Methods

### 2.1. The Seguimiento Universidad de Navarra (SUN) Cohort

**Figure 1:**
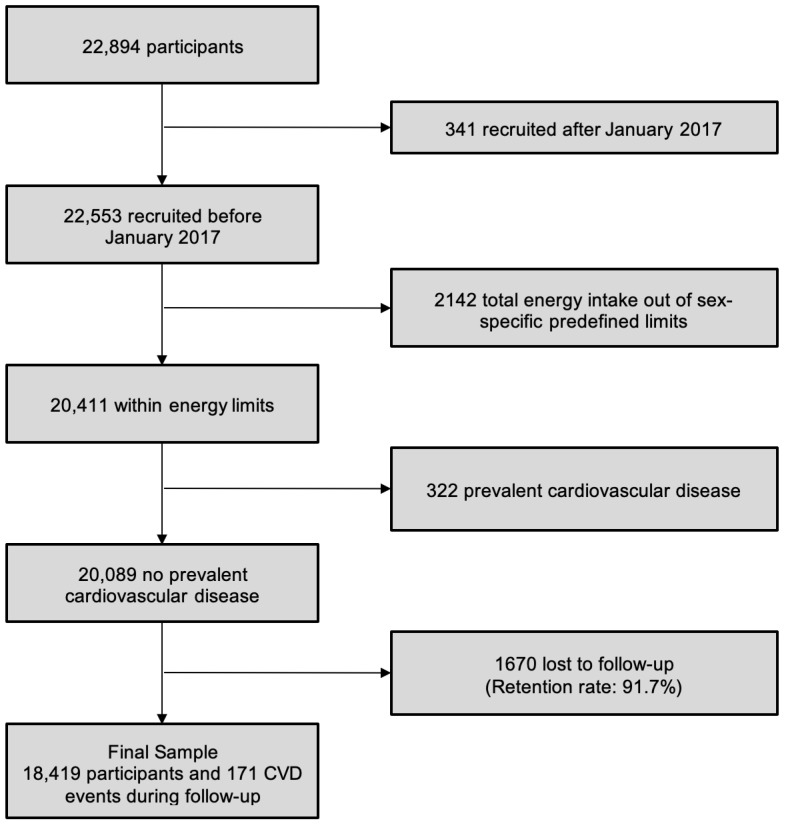
Flow-chart of participants in the SUN cohort in the 2019 data set.

The objectives and methods of the SUN project have been sufficiently reported in detail elsewhere [34,36]. Briefly, this prospective cohort study started in 1999 and enrolled university graduates from all over Spain. More than 50% of participants are health professionals. The SUN cohort is patterned after the models of the Nurses’ Health Study and the Health Professionals’ Follow-up Study, but the recruitment in the SUN cohort is continually open (dynamic design). Participants are followed-up every other year by means of mailed questionnaires. The average age of participants at inception was 38 years and 61% of them were women. Figure 1 shows the flow-chart of participants and the overall retention proportion in the cohort (93%).

### 2.2. Exposure Assessment

The method for dietary assessment consists in a repeatedly validated 136-item semi-quantitative food frequency questionnaire (FFQ), sufficiently described elsewhere [37,38,39]. Energy intake was calculated using the dietary intake collected with this validated self-administered semi-quantitative FFQ. Women with total energy intake <500 kcal/d or >3500 kcal/d and men with <800 kcal/d or >4000 kcal/d were excluded (Figure 1).

We used three alternative definitions for assessing adherence to the MedDiet: the Mediterranean Diet Score (MDS), the modified Mediterranean Diet Score (mMDS) and the Mediterranean Diet Adherence Screener (MEDAS). Questionnaires administered at 4-year, 6-year, 10-year and 14-year follow-up included repeated though limited assessments of dietary habits; and a complete full-length FFQ (136 items) exactly matching the baseline FFQ was collected at 10-year follow-up. 

For some analyses, we used cumulative exposure to the MedDiet by averaging the dietary information collected at baseline and after 10-year follow-up for those participants with sufficiently long follow-up who had completed the 10-year dietary assessment. For other analyses, we updated the dietary exposures using the partial information collected in all these assessments (4-, 6-, 10- and 14-year follow-ups). Specific details of all these questionnaires are publicly available at the website http://medpreventiva.es/bJmSk4 (within the section “Descarga de modelos de cuestionarios”).

The Mediterranean Diet Score (MDS), with a potential range from 0 to 9, was the main assessment of exposure because it is the most commonly used index. It was built following the criteria proposed by Trichopoulou et al. [5]. The ratio of the sum of monounsaturated fatty acid intake to the sum of saturated fatty acids (MUFA:SFA) was considered a beneficial component of the traditional Mediterranean diet. Values of zero or one were assigned to eight components, using sex-specific medians of the sample as cut-off points. When the consumption of 6 postulated beneficial components (MUFA:SFA, fruits and nuts, vegetables–excluding potatoes, legumes, cereals and fish) was above or at the median consumption, participants were assigned a value of one for each beneficial item, and a value of zero otherwise. When the consumption of 2 presumed detrimental components (meat and dairy products) was below the median consumption, participants were assigned a value of one, and a value of zero otherwise. An additional point was given to men consuming from 10 g to 50 g of alcohol per day and to women consuming from 5 g to 25 g. Therefore, the potential range of the MDS is from 0 to 9.

Instead of dichotomizations at the medians, the modified Mediterranean Diet Score (mMDS) used sex-specific tertiles [40,41,42] of consumption (1 for the postulated most detrimental tertile, 2 for the middle tertile and 3 for the most beneficial tertile). The 7 postulated beneficial components were fruits, nuts, vegetables (excluding potatoes), legumes, cereals, fish and olive oil. Therefore, in contrast with the MDS, nuts were now separated from fruits, and olive oil replaced the MUFA:SFA ratio. The 2 postulated detrimental components were again meat/meat products and dairy products. For alcohol, the third tertile (score = 3) was assigned for men consuming from 10 g to 50 g of ethanol per day and for women consuming from 5 g to 25 g. Otherwise they were allocated the lowest tertile (1 point). Consequently, the range of the mMDS was from 10 to 30.

The Mediterranean Diet Adherence Screener (MEDAS) with a potential range from 0 to 14 was developed and validated in the PREDIMED trial [43] and it has been subsequently validated in other countries [44], with relevant findings in the US using metabolomic signatures [45]. It consists of 14 items, including 2 questions on food intake habits characteristic of the MedDiet. One point is given for using olive oil as the principal source of fat for cooking, preferring white meat over red meat; and other 12 questions on food consumption frequency for consuming: (1) ≥4 tablespoons (1 tablespoon = 13.5 g) of olive oil/d; (2) ≥2/d servings of vegetables; (3) ≥3 pieces/d of fruit; (4) <1 serving/d of red or processed meats; (5) <1 serving/d of animal fat; (6) <1 cup/d (1 cup =100 mL) of sugar-sweetened beverages; (7) ≥1 glass/d of red wine; (8) ≥3 servings/wk of legumes; (9) ≥3 servings/wk of fish; (10) <2 servings/wk of commercial pastries; (11) ≥3 servings/wk of nuts; or (12) ≥2 servings/wk of “sofrito”, a traditional sauce of tomatoes, garlic, onion, or leeks sauteed in olive oil.

### 2.3. Outcome Assessment

Through repeated contacts with participants (postal mail, email, and telephone calls), we were able to obtain information on the occurrence of incident disease during the follow-up period, including CVD clinical events. We also gathered information on potentially deceased participants from their next of kin, work associates and the postal system. This allowed us to identify more than 85% of deaths. For the rest of deaths, the National Death Index was checked at least once a year to update vital status and identify causes of death, if unknown. All causes of death were coded using International Classification of Diseases, 10th version based on the data provided by the National Death Index. We excluded participants with short time follow-up (recruited after January 2017).

The primary end-point study was a composite of CVD events including myocardial infarction, stroke, and CVD death, which were defined according to the same criteria used in the PREDIMED trial [4]. We requested the medical records from the participants or their families in the event that any of these diagnoses were reported in any of the follow-up questionnaires. All reported cases were evaluated and confirmed by an expert medical team that did not have prior knowledge of the participants’ dietary or lifestyle information. Participants with CVD at baseline (those having a previous medical diagnosis of major CVD) were excluded from the study.

### 2.4. Potential Confounder Assessment

A thorough collection of information was performed in the baseline assessment (with 554 items) using the enrolment questionnaire. This assessment included sociodemographic information, dietary intake, exposure to active and passive smoking and other substance use habits, anthropometric measurements, medical history, physical activity, exposure to screening practices, frequency of visits to the doctor and other aspects of participants’ lifestyles and cardiovascular risk factors [36]. The methods for collecting anthropometric measurements [46] and physical activity/sedentary lifestyles [47] were previously validated. Body mass index (BMI) was calculated by dividing weight by height squared (kg/m^2^). To assess potential confounding by an overall degree of health consciousness, we developed a composite index considering whether or not the participant routinely underwent 10 types of screening procedures (general medical check-up, coronary stress test, chest X-ray exam, fecal occult blood test, colonoscopy/sigmoidoscopy, dental check-us, intraocular pressure measurements, cervical cancer screening tests, mammography or prostatic specific antigen detection).

### 2.5. Statistical Analyses

A wide array of statistical strategies were employed to reduce the limitations of observational study design and approach causal inference: (1) To account for potential inconsistencies or some variability in MedDiet definitions, as suggested by Zaragoza-Martí et al. [48] and by Abdelhamid et al. [49], we compared whether the results found for the association between baseline adherence to the MDS and CVD events were replicated when adherence to the MedDiet was alternatively appraised with the mMDS or with the MEDAS. All these analyses were conducted with Cox regression models using age as the underlying time variable and applying different degrees of adjustment. In these models, the exit time was the date of last follow-up, date of the first cardiovascular event or date of death, whichever occurred first. Participants who were alive and free of CVD as of the date of last follow-up or were lost to follow-up were considered censored as of the date of last contact. In addition to age (underlying time variable), the fully adjusted model controlled for the following potential confounders: sex, total energy intake (continuous), smoking habits (4 categories), cumulative exposure to smoking (pack-years, as a continuous variable), passive smoking (smoker spouse), diagnosed dyslipidemia at baseline, BMI (continuous, including also a squared term to account for non-linearity), prevalent hypertension at baseline, prevalent diabetes at baseline, family history of premature coronary heart disease, leisure-time physical activity (METs-h/wk, continuous), average daily hours of television watching (continuous), unemployment and an index of health consciousness (range 0 to 10) based on the attendance to screening procedures. In addition, all models were stratified by decades of age, years of attained university studies (5 categories), marital status (5 categories) and period of entrance in the cohort (5 categories). In these models we grouped the MDS in 3 categories and the other 2 MedDiet scores in 4 categories, always leaving the lowest category as the reference group. We also assessed each MedDiet score as a continuous exposure and estimated the effects for an increment of 1 standard deviation (SD). In addition, we used time-dependent Cox models with cumulative exposures (average of the MDS at baseline and year 10 as the relevant exposure) and inverse probability weighting (IPW) [50,51] to control for confounding with weights calculated using the same potential confounders and stratification variables mentioned above. (2) To test whether the association was dependent on only some components of the MedDiet, we reran the described Cox models for each of the 9 components of the MDS. In addition, we removed each of these components one at a time and estimated the hazard ratio (HR) of the resulting score including only 8 components. We evaluated the influence of each of the dietary components on the HR associated with the MDS by subtracting sequentially one component at a time from the original score while estimating the nine mortality ratios associated with a two unit increment in the score minus vegetables, score minus pulses, score minus fruits and nuts, score minus cereals, score minus fish and seafood, score minus lipid ratio, score minus meat and meat products, score minus dairy products, and score minus ethanol. To preserve comparability, we followed the approach previously used by Trichopoulou et al. [52]. (3) In order to assess the potential for unaccounted confounding or residual confounding, we calculated the E-value as proposed by Vanderweele [53], which–despite recent criticism–stands as a valid method to simplify sensitivity analyses when it is interpreted carefully [54]. (4) For the same purpose, we graphically represented the HR of CVD according to baseline adherence to the MDS (both for a 2-point increment and for the comparison between extreme categories) after sequentially (one at a time) controlling for each additional potential confounder, using the same confounders mentioned above. Confounders were progressively included in the model according to the magnitude of the reported associations based on previous knowledge. The rationale for this graph was that should residual confounding be present, the successive estimates would show an attenuation of the effect (i.e., a shift towards the null value), while the progressive departure from the null would speak against residual confounding. (5) To discard the possibility that our analytical sample could be a peculiar cohort and the results could be constrained to particular characteristics of the assessed cohort, we applied a resampling approach to the comparison between extreme categories of the MedDiet: we achieved this by taking 1000 random samples (each of them comprising only fifty percent of the cohort) and reran a Cox model for each of them, with repeated measurements of the MDS and using IPW for controlling all mentioned potential confounders at baseline. We calculated the percentiles 1st, 2.5th, 25th, 50th, 75th, 97.5th and 99th for the distribution of the 1000-point estimates of the HR. We repeated this same resampling procedure with 1000 random samples from each of the 3 strata of age (≤45; 45 to <55; ≥55 years), but randomly selecting 75% of the strata (instead of 50%) to increase statistical power in each of these 3000 within-stratum random samples. (6) To emulate the per-protocol analysis of a hypothetical target trial [55,56,57] analogous to the PREDIMED trial, that compared a MedDiet to a low-fat diet (control group), we assigned participants 55 years or older to a simulated low-fat diet if during follow-up they kept the goal of having a total fat intake below 30% of total calories, or to a simulated MedDiet intervention group if they kept total fat intake above 30% of calories and achieved a MEDAS score (the one used in PREDIMED intervention) ≥ 8. We updated this allocation to the simulated MedDiet intervention group or the simulated low-fat diet control group with repeated follow-up information obtained at 4-, 6-, 10- and 14-year follow-up and used a multivariable-adjusted time-dependent Cox model (with time in trial as the time scale, instead of using age) to estimate the multivariable-adjusted HR of CVD for the intervention group (MedDiet) as compared to the control group. This approach simulated the per-protocol analysis of a pragmatic trial and we censored participants when they stopped adhering to the intended intervention [58]. Our primary focus in this analysis was to assess participants 55 years or older, as the inclusion criteria of the PREDIMED trial imposed. The characteristics of the PREDIMED trial and the emulated trial are summarized in Supplementary Table S1. (7) In order to represent the absolute risks of CVD for three categories of cumulative adherence to the MDS throughout the follow-up time, we represented Nelson–Aalen curves as customarily presented in randomized controlled trials (RCTs), although they were adjusted using IPW with weights calculated according to all the previously mentioned potential confounders at baseline. (8) Finally, to evaluate whether the observed association might be the result of some artifact or another potential bias, we adopted the approach known as negative controls [59,60], and applied the main multivariable-adjusted Cox model with baseline MDS (for a 2-point increment) exposure to assess its association with the following naïve, non-CVD, incident outcomes (i.e., negative controls): undergoing mammography (women) or PSA testing (men); any visit to a doctor; sport injuries; road injuries with hospitalization; road injuries without hospitalization; cataract surgery; incidence of glaucoma and a new diagnosis of bronchitis. Additionally, we calculated the rate advancement period (RAP), which provides a metric to assess the time by which a rate of a specific outcome is advanced (positive values for detrimental exposures) or by which it is postponed (negative values for protective exposures) among exposed subjects compared with unexposed individuals, conditional on having the same levels of the other factors included in the fully adjusted model [61,62]. It is useful to analyze outcomes that uniformly rise with age, as happens with the incidence of CVD.

## 3. Results

Among the 22,894 participants in the SUN cohort in the 2019 data set, we excluded 341 participants (1.5%) with short time follow-up (recruited after January 2017), 2142 (9.4%) who reported energy intake out of sex-specific predefined limits and 322 (1.4%) with prevalent cardiovascular disease at baseline. Moreover, 1670 (7.3%) participants were lost to follow-up, resulting in a retention rate of 91.7%. Thus, the final sample included 18,419 participants (Figure 1). During a mean follow up of 11.5 years, 171 events of cardiovascular disease were blindly adjudicated as confirmed cases by the independent panel of medical doctors of the SUN cohort, who were masked to the dietary habits and lifestyle of participants.

Baseline characteristics according to baseline MDS score are shown in Table 1. Participants with better adherence to the MedDiet were slightly older, with a higher proportion of males, higher total energy intake and physical activity and better health consciousness, but they had a higher burden of cardiovascular risk factors, due to a higher prevalence of dyslipidemia, hypertension, diabetes, former smoking and family history of premature coronary heart disease (CHD).

When the 3 indexes appraising adherence to the MedDiet were considered as continuous variables, we observed that one SD increase in the baseline MDS score was associated with a 29% relative reduction in the risk of CVD (95% CI 14–41%) after adjusting for all potential confounders (Table 2). A stronger association was found when the 2 extreme categories of the MDS score were compared with updated dietary assessments using cumulative averages (HR: 0.30; 95% CI 0.14–0.62 for the comparison of 7–9 points versus 0–2 points in the MDS). The E-value resulted in 6.12 for that estimate, which was higher than any of the HRs obtained for the confounders, except for age (used as the time variable in our analyses). 

Similar, though slightly weaker, associations were found when using the mMDS or the MEDAS (with analysis restricted to participants older than 40 years at baseline). When the adherence to the MedDiet was calculated with the mMDS or the MEDAS indices, participants were classified in 4 categories (Table 2). Compared with participants in the lowest category, those in the third and fourth categories of mMDS score showed significant reductions in the risk of CVD. When the MEDAS was used, that association was only observed for participants over 40 years old. The risk reduction observed for one SD increase was 20% (95% CI 3–44%) for the mMDS and 17% (95% CI 1–31%) for the MEDAS (only in the analysis restricted to participants above 40 years old at baseline).

**Figure 2:**
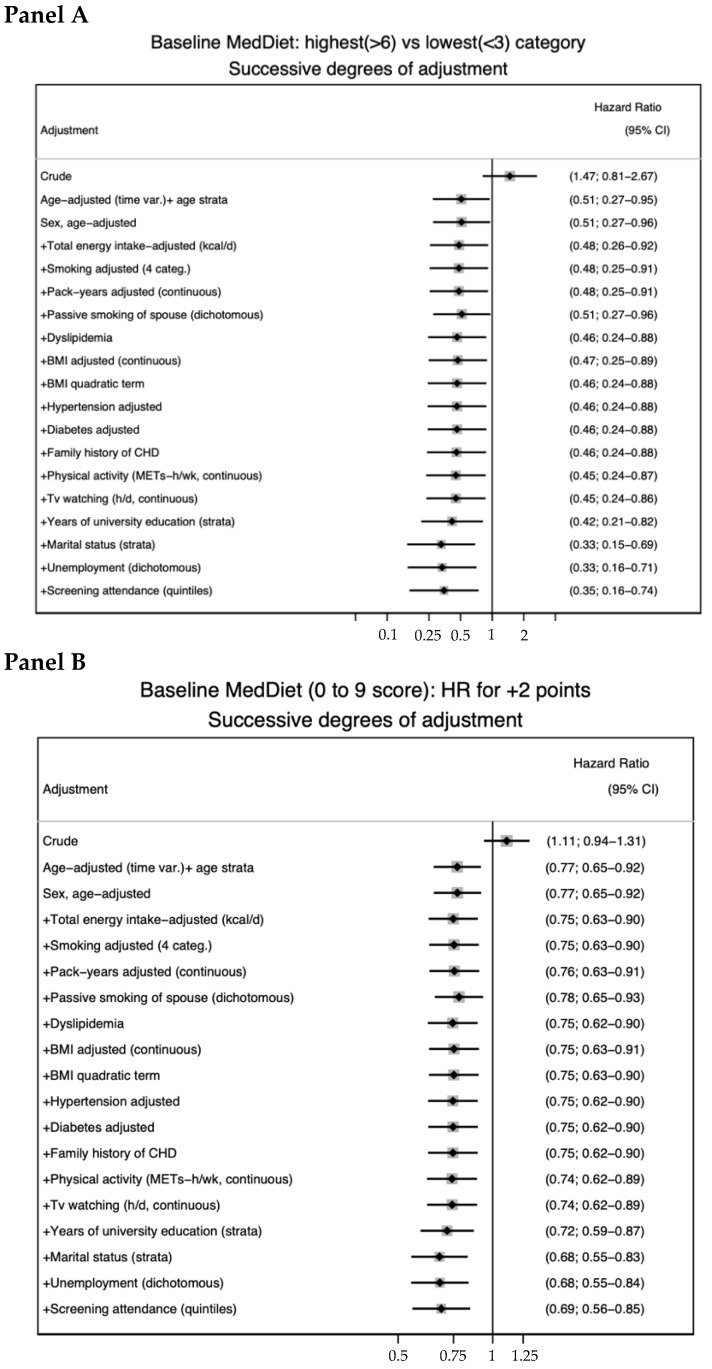
Progressively adjusted HRs for CVD for the comparison between extreme categories (**Panel A**) or associated with 2 additional points in the MDS index (**Panel B**), adding one additional confounder at a time.

Figure 2 shows progressively adjusted HRs for CVD for the comparison between extreme categories (Panel A) or associated with 2 additional points in the MDS index (Panel B), after adjusting for successive potential confounders, adding an additional confounder, one at a time. The greatest change in the HR for every 2 additional points in the MDS was observed from the crude model that did not consider age (HR: 1.11; 95% CI: 0.94–1.31) to the age-adjusted model (HR: 0.77; 95% CI: 0.65–0.92), in which age was the underlying time variable and age strata were included as a stratifying factor. In the comparison between extreme categories of adherence to the MedDiet, the progressively better control for potential confounders also resulted in stronger inverse estimates with an increasing departure from the null as a higher number of covariates were adjusted for.

We found that none of the individual items of the MDS index measured at baseline or updated during follow-up was independently associated with the risk of CVD (Table 3). In further analyses, we calculated the relative change in the HR for CVD associated with the equivalent to a 2-point increase in the baseline MDS after removing one item of the index at a time. The elimination of any of the items resulted in an attenuation in the HR for CVD associated with the index. The most significant change was observed with the elimination of fish and seafood, which resulted in a loss of statistical significance for the index. The next most important change was observed when removing cereals and vegetables. Similar modifications were observed when adherence to the MedDiet was assessed with cumulative averages after updating the MDS in repeated measurements after 10-year follow-up. In that case, the items whose elimination from the index meant greater changes in the HR for CVD were fruits and nuts, followed by cereals, fish and legumes. The removal of any of those items meant an increase of approximately 20% in the HR for CVD associated with the equivalent to a 2-point change in the MDS, but none of them was able to nullify the inverse association when they were removed. These results suggested that all 9 components were important contributors but no single one of them was able to fully account for the inverse association.

When we applied a resampling procedure to estimate HR in 1000 random samples comprising 50% of the cohort, the distribution of point estimates showed a fairly robust picture for the comparison between extreme categories of the MDS (>6 vs. <3), with median HR = 0.29 (percentiles 1st to 99th: 0.11–0.94) (Table 4). Results within each of the age strata, using similar procedures but with samples containing 75% of each age stratum, showed a larger spread for subjects 54 year or younger, but a robust inverse association was found for participants 55 years or older, median HR = 0.31 (percentile 1st to percentile 99th: 0.14 to 0.73) (Table 4).

We emulated the per-protocol analysis of a target trial, using repeated measures of adherence to both the MedDiet and the low-fat diet (updated at 4, 6, 10 and 14 years). The target trial was the PREDIMED trial, the main published trial of MedDiet, which included only subjects aged 55 years or older on a MedDiet versus a low-fat diet [4]. Using this approach, when the MedDiet was compared to the low-fat diet among subjects 55 years or older (n = 2782, including 53 cardiovascular events), we found a multivariable-adjusted HR = 0.38 (95% CI: 0.16–0.91). This point estimate (HR = 0.38) resembled the result of the per-protocol analysis in the published target trial (HR = 0.42) [4].

**Figure 3:**
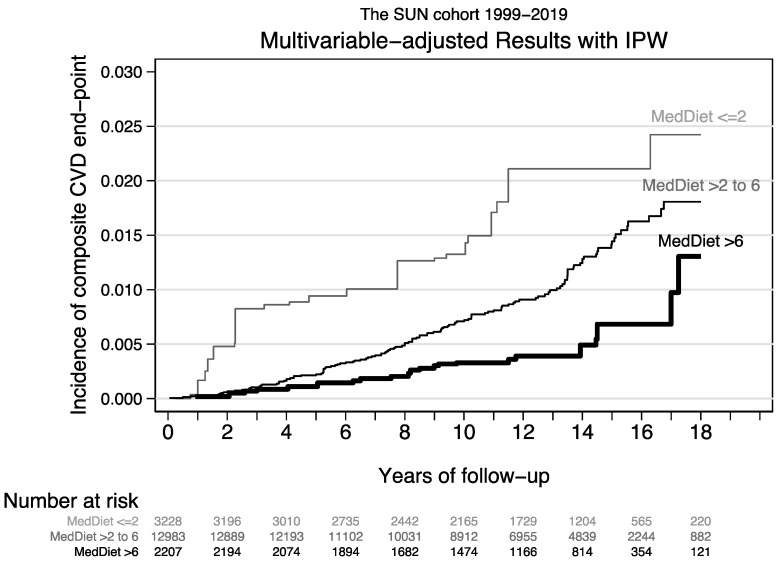
Multivariable adjusted curves of absolute cumulative incidence of CVD according to baseline adherence to the MedDiet (3 categories of the MDS). Confounding was adjusted using IPW methods.

After applying IPW methods to adjust for confounding, curves of absolute cumulative incidence according to baseline adherence to the MedDiet (3 categories of the MDS) were graphically plotted, showing the early departure of the Nelson–Aalen curves after 1 year of follow-up (Figure 3). Multivariable-adjusted absolute cumulative risks at 5-year follow-up were 9.4/10^3^ for low adherence to MedDiet (MDS < 3), 2.2/10^3^ for intermediate adherence (3 ≤ MDS ≤ 6) and 1.1/10^3^ for high adherence (MDS > 6). Respective cumulative absolute risks at 10-year follow-up were 13.2/10^3^ for low adherence, 7.1/10^3^ for intermediate adherence and 3.3/10^3^ for high adherence. The multivariable-adjusted annualized rates over the total observed follow-up period were 1.55 per 10^3^ person-years in the low adherence category, 0.84 per 10^3^ person-years for moderate adherence, and 0.40 per 10^3^ person-years for high adherence. The numbers needed to treat for the comparison between high and low adherence were 120 after five years, 101 after ten years and 870 yearly.

In the analysis using negative controls, no evidence was found that the MedDiet (for each 2 points in the MDS) was associated with a reduced risk of several other naïve and biologically less plausible outcomes (Table 5), and most point estimates for the HRs of these negative controls were very close to the null.

In subgroup analysis, the results for the comparison between extreme categories of baseline adherence to the MedDiet were consistent across categories of age, sex, cardiovascular risk factors, educational level, sociodemographic characteristics, and the index of health consciousness (Supplementary Figure S1).

Finally, as estimated by the RAP, the rates of CVD would be postponed by an average of 7.9 years (95% CI: 1.6 to 14.2 years) by switching from low to high adherence to the MedDiet (RAP = −7.92, 95% CI: −1.61 to −14.22 in the fully adjusted model for the comparison of MDS = 7 to 9 versus MDS = 0 to 2).

## 4. Discussion

We developed a variety of analytical procedures to investigate the causal effect of a potential intervention encouraging adherence to the traditional MedDiet using data from an observational cohort. The advantage of our approach was that the RCT had previously been published and it obtained similar results in its per-protocol analysis to those obtained in our simulation of the comparison of the MedDiet versus a low-fat diet control group. The HR in the per-protocol analyses of PREDIMED trial was 0.42 (95% CI: 0.24–0.63) [4], whereas it was 0.38 (95% CI: 0.16–0.91) in our simulated trial using observational data. Interestingly, both studies used the same adherence score to the MedDiet (MEDAS) [43,44,45].

This is the first time that we present absolute risks of CVD according to adherence to the Mediterranean diet and depict these multivariable-adjusted absolute risks graphically across 18 years of follow-up in the SUN cohort. This cohort was specifically designed to assess the effect of the MedDiet on CVD. The description of absolute risks throughout a long follow-up period—by graphically depicting the natural history of the causal effects of the exposure on the outcome and the estimation of the number needed to treat—are less frequently reported in observational studies than in RCTs. However, as we show here, an appropriate analysis of observational data can provide analogous results to those of a RCT, once the case for causal inference is adequately supported on robust grounds. We estimated that 101 subjects would need to change their dietary habits from low to high adherence to the traditional MedDiet in order to prevent a major severe clinical event of CVD during the subsequent 10 years. This number renders interventions with the MedDiet sufficiently efficient.

The MDS and mMDS are dietary indexes designed to be derived from an FFQ. It is well known that FFQs are not optimal tools for accurately measuring exact dietary intakes, but they are very useful for ranking participants according to their dietary intake. The MEDAS, on the other hand, was designed as a rapid screening and intervention tool for measuring adherence to the Mediterranean diet, but it does not use rankings and cannot capture so well the characteristics of participants in relation to the overall cohort. In this line of thought, we believe that the attenuation of the effect when using MEDAS instead of MDS or mMDS may be due, at least in part, to the intrinsic limitations of MEDAS (designed as a screening/intervention tool), which is not able to capture the reality of the overall dietary intake of each participant by ranking it in the context of the whole cohort.

Our observation was that the greater the number of potential confounders adjusted for, the stronger the inverse association found, which speaks against important residual confounding. Several other analytical procedures used in this report also allay potential criticisms of residual confounding, including the large, estimated E-value [53], such as the ability to adjust for some proxies of health consciousness, the null results in the assessment of naïve negative controls and the consistency of findings under different assumptions and resampling approaches. In addition, the restriction to a fairly homogeneous cohort regarding socioeconomic and educational factors offers an additional protection against potential residual confounding.

The authors of a previous observational study on a dietary pattern (the American Heart Association 2020 (AHA 2020) Dietary Goals) emulated a target trial [57]. They reported that “the AHA 2020 Goals are generally consistent with the Mediterranean diet: both include increased intakes of fruits and vegetables, nuts and legumes, and fish, and limited intakes of sugar-sweetened beverages and processed red meat”. Subsequently they used the PREDIMED trial as a benchmark. The novelty of our approach is that we precisely assessed the MedDiet, and not another dietary pattern, and that we used the same score (MEDAS) that was applied in the PREDIMED trial (our target trial) to conduct the intervention.

We acknowledge that although the threat of residual confounding seemed remote in our results, it cannot be completely discarded in an observational study. We can only conclude that an alternative explanation of findings claiming that it was due to residual confounding appeared to be very unlikely.

Other limitations of our assessment were based on the reduced number of events as was to be expected in a young and healthy cohort. This fact was reflected in the width of some confidence intervals.

The use of questionnaires and the self-reported information provided by participants represents a potential limitation because there might be some degree of measurement error. This possibility also includes the effects of the communication methods (mainly, the use of postal services at the beginning of the cohort, and web-based questionnaires in recent years). However, we previously demonstrated good validity of web-based questionnaires in this cohort and showed that there was not any loss of data quality in this cohort of highly educated adults when using this method of communication [63]. Furthermore, the most likely direction of the effect of measuring errors in exposure assessment would be towards the null.

Losses to follow-up (around 8% in our case) represent the main threat for internal validity in cohort studies. Previously, we quantitatively addressed this issue in the SUN cohort and found reassuring results [64,65]. In addition, the percentage of attritions was not large.

Another potential limitation could be related to the selection of highly educated participants, notably of whom 60.5% were women (since a recent RCT suggested differential effects in men and women) [34]. Admittedly, the SUN cohort cannot be considered as a representative sample of the general Spanish population. This fact may affect generalizability of our reported results. On the other hand, this feature of the design of the SUN cohort (which is common to most epidemiologic cohorts and RCTs) may in fact contribute to reinforcing the internal validity of our findings, because the high level of education and homogeneity of the cohort reduce potential confounding related to socio-economic status and educational level. Moreover, these characteristics make it possible to collect high quality information using questionnaires.

In any case, after comparing the similarity of our results with those of two actually conducted RCTs [4,34], the results found in our observational study do support causality. In addition, there was a confluence of many criteria to support causality (strong inverse associations, biological plausibility, an immense accrual of similar results in cohorts all around the world [7,17], the dose-response pattern, the experimental evidence, and the reasonable absence of unknown/unmeasured strong confounders). This is an argument to moderate the tendency to ban the use of terms such as “causal effects” or other similar expressions in scientific publications that describe nonrandomized studies [29]. There is empirical evidence to support the considerable similarity between the scientific answers obtained with randomized and nonrandomized designs, including nutritional research [28,66]. Instead, the tendency should be to encourage researchers to evaluate different statistical approaches, equal or analogous to the ones presented in this study or other procedures better suited to the studied hypotheses. It should be acknowledged that the aim of observational research in nutritional epidemiology is to achieve causal conclusions and not only to show ‘associations’. In fact, a purely associational goal would not use any sort of adjustment for confounding [29].

Our results also provide a ground for refuting speculative criticisms on the methods used by large and well conducted observational studies on nutritional epidemiology [25,26,27,28]. Major criticisms of these studies were based on the claim that they did not use a randomized procedure to allocate the dietary exposures. However, Schwingshackl et al. already reported a sufficiently high agreement between bodies of evidence from RCTs and observational cohort studies in nutrition research [28], as we also provide here with high specificity for the MedDiet.

## 5. Conclusions

In conclusion, a variety of analytical procedures contributed to support the causal effect of a traditional MedDiet on CVD prevention using observational data. These data can be used to inform health policy and develop health promotion strategies. Despite the always present threat of measurement error and residual confounding, in practice—for ethical and practical reasons—only prospective observational cohorts can be used in most cases to answer relevant questions on the long-term effects of potential dietary interventions.

## Figures and Tables

**Table 1:** Baseline characteristics according to baseline MedDiet adherence (MDS) in the “Seguimiento Universidad de Navarra” (SUN) cohort, 1999–2019.

All Participants	Low MDS (0 to 2)	Moderate MDS (3 to 6)	High MDS (7 to 9)
N	3133	13,038	2248
Female sex (%)	62.1	60.8	56.8
Age	33.9 (10.3)	38.2 (12.1)	43.3 (12.7)
Baseline energy intake (kcal/day)	2192 (594)	2352 (625)	2517 (548)
Body mass index (kg/m^2^)	23.0 (3.5)	23.6 (3.5)	24.0 (3.6)
Physical activity (METs-h/wk)	17.6 (20.4)	21.8 (22.7)	27.3 (26.0)
Television watching (h/d)	1.64 (1.3)	1.61 (1.2)	1.57 (1.1)
Years of university education	5.02 (1.5)	5.05 (1.5)	5.11 (1.5)
Health consciousness (scale 0–10)	3.6 (1.8)	4.0 (1.8)	4.6 (1.9)
Dyslipidemia (%)	11.0	16.9	25.6
Hypertension (%)	7.2	10.5	14.3
Diabetes Mellitus (%)	0.7	1.8	2.8
Family history of CHD (%)	6.5	7.7	9.4
Unemployment (%)	4.6	4.3	2.7
Smoking			
Never smokers (%)	54.9	47.8	41.0
Current smokers (%)	22.6	22.3	19.6
Former smokers (%)	21.7	29.2	38.8
Missing (%)	0.8	0.8	0.7
Pack-years of smoking	3.4 (7.7)	4.8 (9.5)	6.1 (10.6)
Passive smokers (%)	14.4	19.3	22.2
Marital status			
Unmarried (%)	54.8	43.4	33.5
Married (%)	42.4	51.7	59.9
Divorced (%)	1.6	2.5	3.3
Widowed (%)	0.4	0.9	1.6
Others (%)	0.8	1.5	1.7
Year of entering the cohort			
1999–2001 (%)	40.6	33.6	25.1
2002–2003 (%)	22.9	19.0	15.9
2004–2006 (%)	17.0	22.8	28.8
2007–2008 (%)	11.9	15.3	19.6
2009–2016 (%)	7.7	9.2	10.6
Components of the MDS			
MUFA:SFA Ratio	1.1 (0.2)	1.3 (0.3)	1.6 (0.4)
Fruits and nuts (g/day)	185 (142)	357 (287)	551 (367)
Vegetables (g/day)	322 (185)	538 (334)	764 (368)
Cereals (g/day)	74 (59)	103 (73)	134 (73)
Fish (g/day)	64 (39)	100 (60)	135 (59)
Legumes (g/day)	17 (14)	23 (19)	29 (18)
Dairy products (g/day)	298 (222)	186 (189)	84 (100)
Meat (g/day)	197 (80)	175 (79)	145 (67)
Alcohol intake (g/day)	4.0 (8.3)	6.7 (10.3)	9.7 (10.3)

MET: Metabolic equivalent task; CHD: coronary heart disease; MUFA: monounsaturated fatty acids; SFA: saturated fatty acids.

**Table 2:** Hazard ratios (95% confidence intervals) of CVD according to different MedDiet adherence scores in the “Seguimiento Universidad de Navarra” (SUN) cohort, 1999–2019.

**Baseline MDS (range 0 to 9)**	**<3**	**3 to 6**	**>6**		**Per SD**
N	3133	13,037	2248		
Cases	22	127	22		
Person-Years	37,390	149,936	24,604		
Age-, sex-adjusted HR (95% CI)	1 (ref.)	0.92 (0.58–1.45)	0.57 (0.31–1.05)		0.82 (0.70–0.95)
MV-adjusted HR (95% CI)	1 (ref.)	0.83 (0.49–1.41)	0.35 (0.16–0.74)		0.71 (0.59–0.86)
**Modified Mediterranean diet score (10 to 30)**	**<17**	**17 to 19**	**20 to 22**	**23 to 20**	**Per SD**
N	4240	6227	5348	2603	
Cases	39	57	44	31	
Person-Years	50,480	72,274	60,819	28,358	
Age-, sex-adjusted HR (95% CI)	1 (ref.)	0.88 (0.58–1.32)	0.72 (0.46–1.12)	0.77 (0.47–1.24)	0.90 (0.78–1.05)
MV-adjusted HR (95% CI)	1 (ref.)	0.72 (0.45–1.16)	0.58 (0.34–0.97)	0.50 (0.27–0.93)	0.80 (0.66–0.97)
**Mediterranean diet adherence screener** **(MEDAS, 0 to 14)**	**<5**	**5 to 6**	**7**	**8 to 14**	**Per SD**
N	4807	6969	2877	3765	
Cases	36	70	29	36	
Person-Years	57,277	80,741	32,707	41,206	
Age-, sex-adjusted HR (95% CI)	1 (ref.)	1.00 (0.67–1.50)	0.88 (0.54–1.44)	0.68 (0.43–1.09)	0.90 (0.77–1.05)
MV-adjusted HR (95% CI)	1 (ref.)	0.97 (0.63–1.48)	0.85 (0.51–1.43)	0.63 (0.38–1.05)	0.88 (0.75–1.04)
**Mediterranean diet adherence screener (MEDAS, 0 to 14)** **(restricted to >40 years)**	**<5**	**5 to 6**	**7**	**8 to 14**	**Per SD**
N	1354	2634	1283	2032	
Cases	31	65	28	31	
Person-Years	16,005	30,573	14,617	22,636	
Age-, sex-adjusted HR (95% CI)	1 (ref.)	1.00 (0.65–1.54)	0.89 (0.53–1.48)	0.60 (0.36–0.99)	0.86 (0.74–1.01)
MV-adjusted HR (95% CI)	1 (ref.)	0.96 (0.61–1.51)	0.82 (0.47–1.41)	0.53 (0.30–0.91)	0.83 (0.69–0.99)
**MDS cumulative average, time-dependent Cox model with IPW**	**<3**	**3 to 6**	**>6**		**Per SD**
N	3047	13,136	2235		
Person-Years	35,701	151,622	24,855		
Age-, sex-adjusted HR (95% CI)	1 (ref.)	0.93 (0.54–1.61)	0.61 (0.31–1.20)		0.89 (0.74–1.07)
MV-adjusted HR (95% CI)	1 (ref.)	0.65 (0.35–1.21)	0.30 (0.14–0.62)		0.63 (0.43–0.94)

HR: hazard ratio; MDS: Mediterranean diet score; SD: standard deviation; IPW: inverse probability weighting; MV: multivariable adjusted.

**Table 3:** Hazard ratios (95% confidence intervals) for each component of the MDS (yes/no) and effect of removing each component of MDS (one at a time) in the HR of CVD for the equivalent to +2 points in MDS in the “Seguimiento Universidad de Navarra” (SUN) cohort, 1999–2019.

Baseline Exposure	Cases When Item = 0	Cases When Item = 1	Adjusted HR (for Each Item without Adjustment for the Other Items)	*p* Value	Additionally Adjusted for the Other 8 Items, HR (95% CI)	*p* Value
Ratio MUFA:SFA	84	87	0.856 (0.606–1.211)	0.380	0.980 (0.682–1.409)	0.913
Fruit and nuts	76	95	0.714 (0.494–1.031)	0.072	0.797 (0.545–1.164)	0.240
Vegetables	90	81	0.756 (0.528–1.082)	0.126	0.820 (0.566–1.189)	0.296
Cereals	102	69	0.745 (0.510–1.090)	0.130	0.750 (0.507–1.109)	0.149
Fish and seafood	83	88	0.774 (0.542–1.104)	0.157	0.797 (0.555–1.146)	0.221
Legumes	85	86	0.720 (0.501–1.035)	0.076	0.717 (0.497–1.034)	0.075
Low dairy	77	94	0.720 (0.499–1.040)	0.080	0.780 (0.531–1.145)	0.205
Low meat	74	97	0.856 (0.596–1.231)	0.403	0.885 (0.609–1.286)	0.522
Mod. alcohol	113	58	0.955 (0.660–1.381)	0.806	0.949 (0.655–1.376)	0.783
**Repeated measures, cumulative averages**						
Ratio MUFA:SFA	76	95	0.894 (0.632–1.266)	0.528	1.021 (0.709–1.471)	0.910
Fruit and nuts	73	98	0.672 (0.465–0.972)	0.035	0.743 (0.508–1.086)	0.125
Vegetables	84	87	0.849 (0.593–1.217)	0.373	0.917 (0.632–1.330)	0.647
Cereals	99	72	0.762 (0.523–1.108)	0.155	0.764 (0.518–1.127)	0.175
Fish and seafood	73	98	0.770 (0.540–1.100)	0.151	0.796 (0.553–1.145)	0.219
Legumes	87	84	0.725 (0.503–1.044)	0.084	0.716 (0.495–1.038)	0.078
Low dairy	78	93	0.758 (0.525–1.094)	0.139	0.803 (0.547–1.179)	0.263
Low meat	72	99	0.779 (0.542–1.121)	0.179	0.808 (0.557–1.174)	0.263
Mod. alcohol	113	58	1.053 (0.731–1.517)	0.780	1.039 (0.721–1.499)	0.836
TOTAL MDS (For +2 points)			0.712 (0.575–0.882)	0.002		
**Effect of removing one item at a time (baseline exposures)**					% of change	
All items (+2 points)			0.686 (0.555–0.848)	0.001	-	
Removing MUFA:SFA ratio			0.772 (0.643–0.928)	0.006	12.56	
Removing fruits and nuts			0.806 (0.673–0.965)	0.019	17.38	
Removing vegetables			0.829 (0.694–0.991)	0.040	20.83	
Removing cereals			0.843 (0.717–0.992)	0.040	22.87	
Removing fish and seafood			0.852 (0.717–1.013)	0.069	24.17	
Removing legumes			0.805 (0.684–0.947)	0.009	17.33	
Removing low dairy			0.781 (0.651–0.936)	0.008	13.77	
Removing low meat			0.797 (0.674–0.942)	0.008	16.16	
Removing alcohol			0.796 (0.677–0.937)	0.006	16.05	
**Effect of removing one item at a time** **(repeated measures, cumulative averages)**					% of change	
All items (+2 points)			0.712 (0.575–0.882)	0.002		
Removing MUFA:SFA ratio			0.835 (0.750–0.930)	0.001	17.22	
Removing fruit and nuts			0.862 (0.777–0.958)	0.006	21.04	
Removing vegetables			0.849 (0.766–0.942)	0.002	19.18	
Removing cereals			0.865 (0.783–0.956)	0.004	21.41	
Removing fish and seafood			0.862 (0.779–0.954)	0.004	21.00	
Removing legumes			0.872 (0.790–0.962)	0.007	22.35	
Removing dairy			0.853 (0.769–0.947)	0.003	19.78	
Removing meats			0.861 (0.778–0.952)	0.004	20.79	
Removing alcohol			0.846 (0.766–0.934)	0.001	18.68	

HR: hazard ratio; MDS: Mediterranean diet score; MUFA: monounsaturated fatty acids; SFA: saturated fatty acids.

**Table 4:** Distribution of the hazard ratios (point estimates) in a resampling analysis with 1000 random samples (each of them comprising only 50% of the full cohort) and in another 1000 random samples within each age stratum (each of them comprising only 75% of that stratum), obtained from the Cox model with repeated measurements of the MDS (>6 vs. <3 points), using IPW for controlling potential confounders.

	Median HR	P 1	P 2.5	P 25	P 75	P 97.5	P 99
All (samples: 50%)	0.29	0.11	0.14	0.22	0.39	0.82	0.94
Age strata (samples: 75%)							
≤45 years	0.44	0.07	0.14	0.32	0.58	1.13	1.41
>45 & <55 years	0.25	0.06	0.08	0.18	0.42	2.41	--
≥55 years	0.31	0.14	0.16	0.25	0.39	0.67	0.73

P: Percentile.

**Table 5:** Negative controls: HR (95% confidence intervals) for every 2 additional points in the baseline MDS in the “Seguimiento Universidad de Navarra” (SUN) cohort, 1999–2019.

	Multivar-adj. HR (95% CI)	*p* Value
Main outcome		
Cardiovascular disease	0.686 (0.555–0.848)	0.001
Negative controls		
Mammography (women) or PSA (men)	0.988 (0.954–1.024)	0.510
Visit to doctor	0.970 (0.931–1.011)	0.149
Sport injury	0.998 (0.959–1.039)	0.937
Road injury with hospitalization	0.980 (0.818–1.174)	0.829
Road injury without hospitalization	1.027 (0.965–1.092)	0.404
Cataract surgery	1.091 (1.003–1.188)	0.043
Glaucoma	1.005 (0.870–1.162)	0.944
Bronchitis	0.946 (0.820–1.091)	0.442

HR: hazard ratio; MDS: Mediterranean diet score; PSA: prostate-specific antigen.

## Data Availability

We will be happy to provide access to the SUN cohort dataset (including data dictionaries), making possible the replication of the main analyses used for the present article. Due to the restrictions imposed by the Informed Consent and the Institutional Review Board, bona fide investigators interested in analyzing the dataset used for the present article may submit a brief proposal and statistical analysis plan to the corresponding author. Upon approval from the SUN cohort Steering Committee and Institutional Review Boards, the data will be made available to them using an onsite secure access data enclave.

## Supplementary Materials

The following supporting information can be downloaded at https://www.mdpi.com/article/10.3390/ijerph192013653/s1, Figure S1: Subgroup analysis: Hazard Ratio (HR) and 95% Confidence Interval (CI) of cardiovascular disease for the comparison between extreme categories of baseline adherence to the MedDiet, Table S1: Characteristics of the PREDIMED trial and the emulated trail with participants in the SUN cohort.
