# Supplementary material for: Mediterranean Diet and Cardiovascular Prevention: Why Analytical Observational Designs Do Support Causality and Not Only Associations
Source: Int J Environ Res Public Health. 2022 Oct 21;19(20):13653. doi: 10.3390/ijerph192013653 (PMC9603524; doi:10.3390/ijerph192013653)
Supplement: Supplementary file 1 [file ijerph-19-13653-s001.zip › ijerph-1957557-Supplementary Table S1.pdf]

Supplementary Table S1. Characteristics of the PREDIMED trial and the emulated trail with participants in the SUN cohort.

	Target trial: PREDIMED trial	Emulated trial with participants in the SUN cohort
**Elegibility criteria**	Men (55 to 80 years of age) or women (60 to 80 years of age) with no cardiovascular disease at enrollment, who had either type 2 diabetes or at least three of the following major risk factors: smoking, hypertension, elevated low-density lipoprotein cholesterol levels, low high-density lipoprotein cholesterol levels, overweight or obesity, or a family history of premature coronary heart disease.	Participants (men and women) in the SUN cohort who were 55 years or older at baseline with no prevalent cardiovascular disease
**Treatment strategies**	Control: Low fat diet explained in a leaflet (<30% of energy intake from fat).<br>Intervention: education to upgrade adherence to the Mediterranean diet using the MEDAS score as the main tool. Participants in the group assigned to a Mediterranean diet received extra-virgin olive oil received (1 l/wk per household), with the recommendation to consume at least 4 tablespoons per day (one of the 14 items in the MEDAS score). Participants in the group assigned to a Mediterranean diet with nuts received 30 g of mixed nuts per day per person (15 g of walnuts, 7.5 g of hazelnuts, and 7.5 g of almonds) at no cost. In the per protocol analysis both groups olive oil+nuts) were merged together.<br>No total calorie restriction was advised, nor was physical activity promoted.	Control: Low fat diet (<30% of energy intake from fat).<br>Intervention: Adherence to the Mediterranean diet was assessed with the MEDAS index.<br>Total calorie intake and physical activity were not considered for the definition of the treatment strategies
**Assignment procedures**	Participants were randomly assigned, in a 1:1:1 ratio, to one of three dietary intervention groups: a Mediterranean diet supplemented with extra-virgin olive oil, a Mediterranean diet supplemented with nuts, or a control diet.	Control: Participants with fat intake below 30% at baseline and during follow-up were included in this group.<br>Intervention: Participants with ≥8 points in the MEDAS score at baseline and during follow-up were included in this group.

	A computer-generated random-number sequence provided randomization tables with four strata (men <70 years of age, men ≥70 years of age, women <70 years of age, and women ≥70 years of age) and were initially generated for 1000 participants (250 per stratum) for each site. A small subset of participants underwent cluster randomization in small clusters (households or, only in one of the 11 centers, small groups in some clinics).	
**Outcomes**	A composite of myocardial infarction, stroke, and CVD death. Four sources of information were used to identify end points: repeated contacts with participants, contacts with family physicians, a yearly review of medical records, and consultation of the National Death Index. All medical records that were related to end points were examined by the end-point adjudication committee, whose members were unaware of the intervention-group assignments. Only end points that were confirmed by the adjudication committee and that occurred between June 25, 2003, and December 1, 2010, were included in the analyses.	A composite of myocardial infarction, stroke, and CVD death.<br>Information was obtained from medical records from the participants or their families in the event that any of these diagnoses were reported in any of the follow-up questionnaires. All reported cases were evaluated and confirmed by an expert medical team that did not have prior knowledge of the participants dietary or lifestyle information. We also gathered information on potentially deceased participants from their next of kin, work's associates and the postal system. For the rest of deaths, the National Death Index was checked at least once a year to update vital status and identify causes of death, if unknown.
**Follow-up**	Control group: Participants in this group received during the first 3 years of the study a leaflet explaining the low-fat diet. Thereafter, they received personalized advice and were invited to group sessions with the same frequency and intensity as those in the Mediterranean-diet groups, with the use of a separate 9-item dietary questionnaire.<br>Intervention group: Dietitians held individual and group dietary-training sessions at the baseline visit and quarterly thereafter. In each session, participants completed the MEDAS questionnaire to assess adherence to the Mediterranean diet.	Participants completed a self-reported questionnaire every other year.<br>Dietary information was updated at 4, 6, 10 and 14 years of follow-up.

**Causal contrast of interest**	Per protocol analysis.	Per protocol analysis.
**Statistical methods**	Cox model with robust variance estimators to account for intracluster correlations in all Cox models, considering as clusters the members of the same household.<br><br>The main analysis was stratified according to site, sex, and educational level; to account for potential imbalances in baseline risk factors among the intervention groups, the model included nine other baseline variables as covariates. This model was also adjusted for propensity scores that used 30 baseline variables to estimate the probability of assignment to each of the intervention groups.<br><br>In the per protocol analysis the Mediterranean diet was compared with the control diet that would have been observed if all the participants had adhered to their assigned interventions throughout the follow-up period. Data for participants when censored when they first stopped adhering to their assigned intervention, inverse-probability weights were estimated to adjust for postrandomization prognostic factors, and the hazard ratio was estimated for an end-point event in the Mediterranean-diet groups (merged together) as compared with the low-fat diet group.	Multivariable-adjusted time-dependent Cox model with time in trial as the time scale, instead of using age.<br><br>This model was also adjusted for propensity scores that used the following baseline variables to estimate the probability of assignment to each of the intervention groups: sex, total energy intake, smoking habits, cumulative exposure to smoking, passive smoking, diagnosed dyslipidemia at baseline, BMI, prevalent hypertension at baseline, prevalent diabetes at baseline, family history of premature coronary heart disease, leisure-time physical activity, average daily hours of television watching, unemployment and an index of health consciousness based on the attendance to screening procedures. In addition, all models were stratified by decades of age, years of attained university studies, marital status and period of entrance in the cohort.
