# Supplementary figures and images for: Mediterranean Diet and Cardiovascular Prevention: Why Analytical Observational Designs Do Support Causality and Not Only Associations
Source: Int J Environ Res Public Health. 2022 Oct 21;19(20):13653. doi: 10.3390/ijerph192013653 (PMC9603524; doi:10.3390/ijerph192013653)

# Subgroup Analysis

High(>6) vs Low(<3) adherence to MedDiet

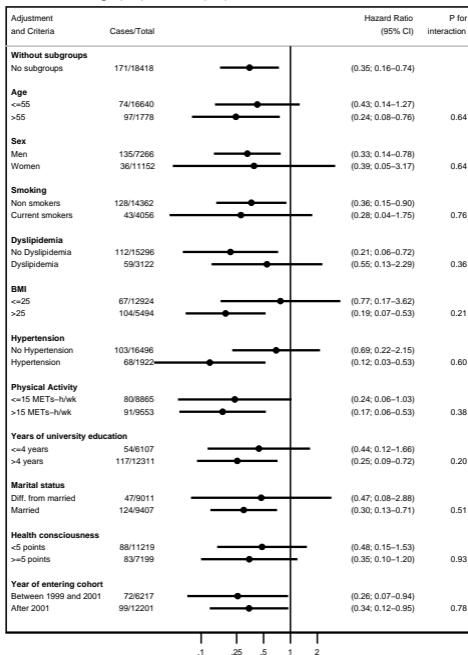

Supplement: Supplementary file 1 [file ijerph-19-13653-s001.zip › ijerph-1957557-Supplementary Figure S1.pdf]
